# A comparative study of four large language models in treatment decision-making for Hip impingement syndrome based on real-world data

**DOI:** 10.3389/fspor.2026.1775462

**Published:** 2026-06-19

**Authors:** Huayu Fan, Zebing Ma, Yongxin Ma, Linlin Wang, Zhifeng Pang, Zhiyuan Cao, Liyun Liu

**Affiliations:** 1 Graduate School, Henan University of Chinese Medicine, Zhengzhou, China; 2 Department of Hip Injuries No. 2, Luoyang Orthopedic Hospital of Henan Province, Orthopedic Hospital of Henan Province, Zhengzhou, Henan, China; 3 Graduate School, Hunan University of Chinese Medicine, Changsha, China; 4 School of Nursing (Nursing School of Smart Healthcare Industry), Henan University of Chinese Medicine, Zhengzhou, China; 5 School of Mathematics and Statistics, Henan University, Kaifeng, China

**Keywords:** AI, artificial intelligence, clinical decision support, femoroacetabular impingement, large language models

## Abstract

**Background:**

Large language models (LLMs) hold potential value in medical decision support, yet empirical studies systematically comparing multiple models for femoroacetabular impingement (FAI) within Chinese clinical contexts remain scarce.

**Objective:**

This study aimed to compare the classification performance, decision consistency, and decision confidence of four mainstream LLMs (GPT-5, Gemini 2.5 Pro, DeepSeek-R1, Grok-4) in FAI treatment decision-making tasks for FAI based on real-world hospitalized cases, and to evaluate their clinical adaptability and limitations.

**Methods:**

We retrospectively included 26 hospitalized FAI cases from our institution (13 surgical group, 13 conservative group), all of which had ethically approved and informed consented. Case information was standardized into two input formats: structured radiology reports only (Group A), and structured radiology reports combined with structured medical records—referred to as multi-source structured text input in this study (Group B). Under both conditions, four LLMs were tasked with providing binary treatment decisions (“surgical” or “conservative”) and indicating decision confidence on a scale from 0 to 100%. Primary evaluation metrics included accuracy, precision, sensitivity, specificity, F_1_ score, and Cohen's kappa. Spearman correlation analysis was used to examine the relationship between LLM decision confidence and accuracy. A hierarchical analysis process (AHP) was employed to derive composite scores through multi-criteria weighting, enabling comprehensive comparison of LLM performance.

**Results:**

Under Group B conditions (incorporating structured medical record information), GPT-5 demonstrated optimal performance, with accuracy of 88%, precision of 92%, sensitivity of 85%, specificity of 92%, F_1_ score of 0.88, and kappa of 0.77, indicating high alignment between its decisions and real-world outcomes. Under the same conditions, the other models showed inferior performance (Gemini 2.5 Pro: 62% accuracy; DeepSeek-R1: 58%; Grok-4: 42%). Across both input modes, GPT-5 exhibited a significant positive correlation between decision confidence and accuracy (Group A: Spearman's *r* = 0.54; Group B: *r* = 0.55, both *P* < 0.01). The confidence-accuracy correlations for the other models were inconsistent and unstable. In the AHP-based composite scoring, GPT-5 achieved the highest Group B score (0.79) and ranked first overall. Overall results indicate that integrating structured radiology reports with structured medical record information (i.e., multi-source structured text information, which simulates a multimodal approach in this study) significantly enhances LLM performance in FAI treatment decision support tasks.

**Conclusion:**

In this exploratory, retrospective single-center study with a small sample size, GPT-5 outperformed the other evaluated models in FAI treatment decision support tasks based on Chinese structured clinical information and demonstrated, to some extent, calibrated decision confidence. These preliminary findings suggest the feasibility of LLMs in orthopedic decision support, however, validation in larger, multi-center, prospective studies is required before broader clinical application. Future studies should adopt multi-center, prospective designs to enhance evaluations of model reproducibility, interpretability, and clinical safety, thereby further validating broader applicability.

## 1 Introduction

In recent years, large language models (LLMs) have been increasingly applied in the medical field, demonstrating significant potential in areas such as assisting with diagnostic and treatment decisions, integrating medical information, and supporting clinical reasoning. Represented by ChatGPT, the new generation of LLMs, with their robust natural language understanding and generation capabilities, are gradually being introduced into clinical workflows to enhance diagnostic and treatment efficiency and decision consistency. In orthopedics, LLM applications have expanded to triage support, preliminary imaging assessment, clinical decision assistance, and resident training (1–4), with studies predicting continued growth in related research (5). Despite this growing research and interest in research and application in orthopedics, exploration of emerging subspecialty conditions remains limited. Femoroacetabular impingement (FAI) is one such condition where mainstream LLMs show significant variations in knowledge architecture, reasoning logic, and comprehension of the Chinese medical contexts. Systematic research and robust evidence remain scarce regarding the actual performance and adaptability of different LLMs. More critically, systematic comparative studies and empirical data specific to the Chinese population and its unique healthcare environment are lacking for emerging conditions like FAI, making it challenging to determine which model is most suitable.

FAI, also known as hip impingement syndrome, is a functional hip disorder caused by abnormal anatomical contact between the acetabular rim and the femoral head/neck region (6). Based on morphological characteristics, it is classified into three types: cam, pincer, and mixed (7). This condition predominantly affects young and middle-aged individuals, particularly those with active lifestyles. Typical manifestations include hip pain and restricted mobility, which significantly impair quality of life. Current treatment approaches primarily encompass conservative management and arthroscopic hip surgery (8). However, selecting the appropriate treatment plan requires careful consideration of multidimensional factors, such as age, activity level, and functional demands, resulting in considerable subjectivity in clinical decision-making. Therefore, clinicians often face inconsistent decisions owning to individual differences in experience when managing such cases, which increases decision complexity and potentially affects treatment continuity and long-term efficacy. Consequently, there is an urgent need to introduce artificial intelligence technology to assist in formulating more standardized, scientific medical decisions.

At present, LLMs have achieved significant results in traditional orthopedic fields such as joint replacement and fracture imaging analysis. However, the potential for FAI assisted diagnosis and treatment support, particularly in the Chinese language context, remains to be thoroughly explored and systematically evaluated. Notably, the high-risk population for FAI—primarily middle-aged and young adults, as well as physically active individuals—overlaps significantly with the primary user base of AI tools. In practice, such patients frequently consult online platforms or LLMs with questions like: “Can hip impingement be managed without surgery?” “Does hip impingement require surgery?” and “How should hip impingement be treated?”. However, due to the fragmented and unsystematic nature of patient inquiries, LLMs often exhibit “hallucination” phenomena in their responses—such as incomplete information or inconsistent logic—resulting in decision outputs of limited reference value that may mislead or cause anxiety among patients. Although medical professionals can provide accurate, comprehensive information post-diagnosis, many patients still prefer self-consultation via the internet or LLMs before seeking medical care. However, the quality of medical information varies widely, and its accuracy is difficult to guarantee. This further highlights the need for systematic evaluation of the performance and suitability of LLMs for professional FAI question-answering scenarios. However, a review of the existing literature reveals persistent research gaps: there remains a scarcity of systematic, multi-model comparative studies grounded in real-world, structured clinical data within the Chinese healthcare context, specifically for emerging conditions like FAI. This lack of empirical evidence hinders a comprehensive understanding of model performance disparities, the relationship between decision confidence and accuracy, and the impact of multimodal information integration.

Against this backdrop, this study aims to compare the performance of four mainstream LLMs—in assisting FAI treatment decisions based on real inpatient clinical cases (GPT-5, OpenAI API; Gemini 2.5 Pro, Google AI Studio; DeepSeek-R1, DeepSeek API; Grok-4, xAI API). It further evaluates their strengths, limitations, and clinical applicability. The specific objectives of this study are as follows:

1. Construct a representative FAI clinical case repository as a standard reference.
2. Quantitatively evaluate the models' binary decision outputs (“surgical or conservative”) and their 0%–100% confidence levels under two information input conditions (structured radiology reports only vs. radiology reports combined with structured medical history).
3. A comprehensive score derived from multidimensional metrics—accuracy, precision, sensitivity, specificity, F_1_ score, Cohen's kappa, and decision confidence-accuracy correlation—weighted by the Analytic Hierarchy Process (AHP), and systematically compare the overall performance and clinical applicability of LLMs using AHP.

## 2 Methods

### 2.1 Medical record screening

#### 2.1.1 Data sources

Cases were enrolled from patients hospitalized at Luoyang Orthopedic Hospital (Henan Orthopedic Hospital) between June 2023 and June 2025 with femoroacetabular impingement (FAI) as the primary or chief diagnosis. All participants provided informed consent, and the study received approval from institutional ethics committee (Approval No.: 2025KYKT0023-02). Cases were categorized into the conservative management group and the surgical group based on treatment protocols administered during hospitalization. Patient admission records were extracted via the hospital information system (HIS) to construct a baseline data table containing age, gender, disease duration, affected side, BMI, and health insurance payment type for comparative analysis. No statistically significant differences were found between groups at baseline, indicating comparability (see Table 1).

**Table 1:** Comparison of baseline data between the surgical group and the conservative management group.

Item	Surgical Group (*n* = 13)	Conservative Management Group (*n* = 13)
Age	19–57	19–59
Age (years)	33.41 ± 5.69	34.41 ± 5.32
Gender (cases) (Male/Female)	6 (46.2%)/7 (53.8%)	8 (61.5%)/5 (38.5%)
Duration of illness (months)	22.16 ± 4.54	23.79 ± 4.89
Affected Side (Left/Right)	4 (30.8%)/9 (69.2%)	6 (46.2%)/7 (53.8%)
BMI (kg/m^2^)	22.01 ± 2.87	22.54 ± 2.45
Health Insurance Payment Type (Type A/Type B)	7 (53.8%)/6 (46.2%)	4 (30.8%)/9 (69.2%)

Type A refers to employee medical insurance, while Type B refers to resident medical insurance.

#### 2.1.2 Case inclusion criteria

1. Medical records must be structurally complete with no significant omissions, and all records must have undergone review by a level 3 physician;
2. The primary or chief diagnosis for hospitalization was FAI;
3. Complete unilateral hip MRI imaging reports were reviewed by at least two physicians;
4. Age range was between 18 and 60 years;
5. The included MRI examinations must have been completed during hospitalization or within 7 days of an outpatient examination.

#### 2.1.3 Exclusion criteria include

1. The physical examination lacked characteristic clinical signs of FAI;
2. Patients with multiple hospitalizations for whom where the current admission could not be definitively attributed to the same disease course;
3. Presence of severe underlying conditions or complications that could significantly impact surgical or conservative treatment decisions;
4. Low-quality medical records (with incomplete descriptions of symptoms or key examinations) rendered the records irreproducible or incapable of standardized processing.

**Figure 1:**
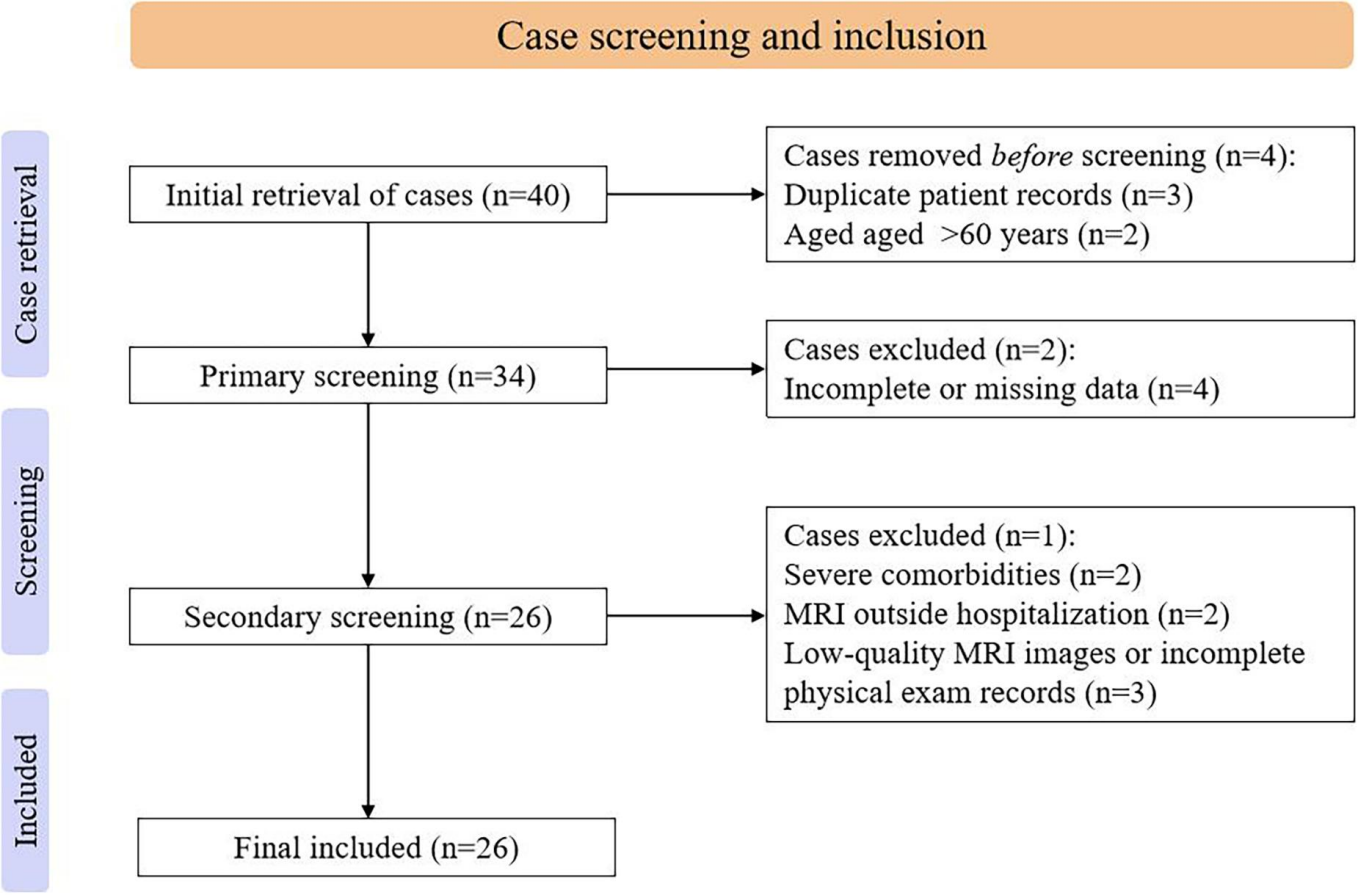
Case screening process

All medical records were independently reviewed by two senior physicians (chief physicians or associate chief physicians) not involved in the patients' initial diagnosis and treatment to confirm compliance with FAI diagnostic criteria. Disagreements during review were arbitrated by a third senior physician. Initial screening yielded 40 cases, which were progressively filtered and excluded, ultimately resulting in 26 cases being included for analysis (see Figure 1).

### 2.2 Data standardization

To minimize heterogeneity in formatting and terminology across the attending physicians and radiologists in medical records and MRI reports, this study systematically standardized the included cases. Following similar LLM studies (9), free-text medical records were organized into predefined structured medical record texts and structured imaging report texts, including fields such as age, gender, occupation, chief complaint, specialty, physical examination, treatment feedback, past medical history, and insurance type. To avoid potential label leakage, the structured input data were constructed exclusively from pre-treatment clinical information, including initial consultation records, imaging reports, and documentation of prior treatment before the current admission. No post-decision or intraoperative information (e.g., surgical notes, postoperative outcomes) was included in the input provided to the LLMs.

Specific standardization criteria included: standardizing current symptoms using the NIH HEAL Pain CDEs (10); referencing physical examination descriptions from the NASS standardized structured medical record; standardizing treatment feedback based on patient-reported outcome measures (PROs); organizing past treatment history using the ICD-10 coding system; and standardizing patient economic status, family support, and occupational background based on the Social Determinants of Health (SDOH) model (11). Additionally, RSNA Rad Report, MRI report texts were structured according to recommendations, extracting and standardizing five primary radiographic features: hip joint alignment and joint space, femoral head morphology, acetabular rim morphology, acetabular labrum morphology and signal, and hip joint effusion. This approach minimized the impact of terminology variations on LLM comprehension (12). Two independent researchers performed the data standardization. Inter-rater discrepancies were resolved by a third researcher, and all standardized data underwent a secondary review by a senior clinical researcher to ensure accuracy and completeness. The task was defined as treatment decision-making. The reference standard for evaluating LLM decisions was the actual treatment administered to the patient during hospitalization, as documented in the final treatment plan. This label (surgical or conservative) was retrospectively assigned based on the patient's medical records and served as the ground truth for all subsequent performance comparisons.

### 2.3 Evaluation of LLMs

This study compared four leading LLMs: GPT-5 (OpenAI), Gemini 2.5 Pro (Google/DeepMind), DeepSeek-R1 (DeepSeek), and Grok-4 (xAI). Brief model characteristics are provided to contextualize their theoretical capabilities: GPT-5 is OpenAI's recently released multimodal LLM emphasizing enhanced reasoning and multitasking capabilities (13); Gemini 2.5 Pro, developed by Google/DeepMind, excels in multimodal data processing and complex reasoning (14); DeepSeek-R1 is an open-source model developed by DeepSeek, employing a mixture-of-experts architecture with chain-of-reasoning capabilities, suitable for generating diagnostic hypotheses and treatment recommendations (15); Grok-4 is a next-generation model launched by xAI, also applicable to medical data analysis (16). To avoid researcher bias, the four models were coded as LLM1 (GPT-5), LLM2 (Gemini 2.5 Pro), LLM3 (DeepSeek-R1), and LLM4 (Grok-4) in the experiments.

### 2.4 Prompt engineering

The core design principle of the prompt engineering was to simulate LLMs as physicians required to make medical decisions (17), enabling them to generate clinical judgments based on input information. Depending on the input format, the approach using only structured imaging reports was designated as Group A, while the approach incorporating both structured imaging reports and structured medical records was designated as Group B. The prompt framework design was as follows: LLMs must generate a decision outcome of “surgical treatment” or “conservative treatment” based on the provided structured medical record information and structured MRI information. Additionally, they are required to provide a decision confidence score ranging from 0% to 100%, categorized into four levels: 0%–25% (high uncertainty), 26%–50% (low confidence),51%–75% (moderate confidence), and 76%–100% (high confidence). This approach to quantifying LLM decision confidence borrowed from the evidence grading framework (18). Prior to formal implementation, the prompt design underwent multiple rounds of optimization and expert validation to ensure it accurately guided LLMs to generate treatment decisions aligned with clinical practice.

The final prompt template was strictly standardized and applied identically to each case across all four LLMs. To account for potential variability, each case was run through each model twice. It was worth noting that the results of the two runs were consistent.

### 2.5 Outcome measures

This study statistically analyzed the decision outcomes generated by different LLMs. Key evaluation metrics include accuracy, precision, sensitivity, specificity, and F_1_ score. Based on these, SPSS 22.0 was used to calculate each LLM's Cohen's kappa value for decision-making tasks, measuring the consistency between LLM outputs and actual clinical decisions. The kappa value ranged from −1 to 1, with values closer to 1 indicating higher consistency. A *P* value of <0.05 indicated statistically significant consistency (19).

The Analytic Hierarchy Process (AHP) was employed to assign weights to multiple indicators and calculate a composite score, enabling the ranking of overall model performance. Weighting was based on the clinical principle of “prioritizing the avoidance of missing patients requiring surgery” in FAI treatment decisions, while referencing common weight distributions in related diagnostic AI studies (20, 21). Accordingly, sensitivity was assigned the highest weight (0.30) as it directly reflects the model's ability to correctly identify surgical or conservative candidates. Accuracy (0.25) and precision (0.20) were weighted next, representing overall and positive predictive performance. The F1 score (0.15) served as a combined measure of precision and recall. Cohen's Kappa (0.05) and specificity (0.05) were assigned lower weights as supplementary indicators for consistency and the identification of non-surgical cases, respectively. This weight distribution was reviewed and agreed upon by the clinical co-authors to ensure clinical relevance.

In addition, based on the specific values of LLM decision confidence, Spearman correlation coefficients were calculated using SPSS software to test the correlation between LLM decision confidence and accuracy. The numerical range from 0 to 1 indicates a positive correlation, and from 0 to −1 indicates a negative correlation, with *P* < 0.05 indicating that the correlation test was statistically significant (22).

## 3 Results

This study included 26 FAI clinical records, of which 13 ultimately underwent surgical treatment, forming the surgical treatment group, and 13 received conservative treatment. A systematic evaluation of the performance of the four LLMs under two information input modes (i.e., Group A and Group B) yielded the following results.

### 3.1 Comparison of classification performance among different LLMs

**Figure 2:**
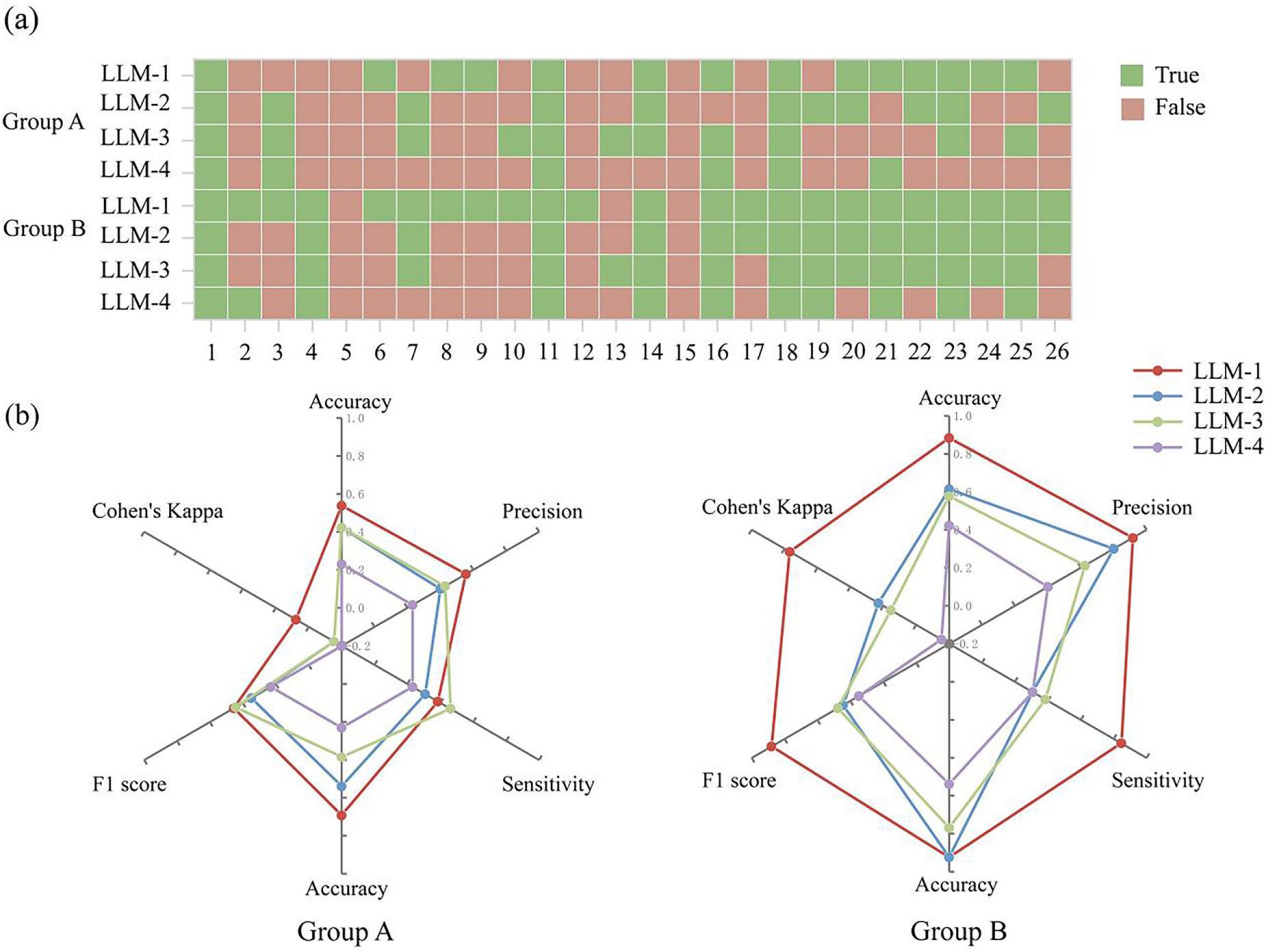
Performance metrics of LLMs under different input conditions. (**a**) Heatmap of true/false positive and negative predictions for across the 26 cases. (**b**) Radar charts comparing accuracy, precision, sensitivity, F1 score, and Cohen's kappa for all four LLMs under Group A and Group B input conditions.

See Table 2 and Figure 2.

**Table 2:** Summary of performance metrics for LLMs under different input conditions.

Model group	Accuracy	Precision	Sensitivity	Specificity	F_1_ Score
LLM1-A	0.54	0.56	0.38	0.69	0.45
LLM1-B	0.88	0.92	0.85	0.92	0.88
LLM2-A	0.42	0.4	0.31	0.54	0.35
LLM2-B	0.62	0.8	0.31	0.92	0.44
LLM3-A	0.42	0.43	0.46	0.38	0.44
LLM3-B	0.58	0.63	0.38	0.77	0.48
LLM4-A	0.23	0.23	0.23	0.23	0.23
LLM4-B	0.42	0.4	0.31	0.54	0.35

### 3.2 Comparison of true/false positive identification and decision consistency

In terms of true/false positive identification, GPT-5 identified 11 true positives (TP) in Group B, misclassified 1 case as a false positive (FP), missed 2 cases (FN), correctly excluded 12 true negatives (TN), resulting in a Kappa value of 0.77 (*P* < 0.01). This indicates a high level of consistency between model outputs and actual medical records. Other models' performance in Group B was as follows: Gemini 2.5 Pro: TP = 4, FP = 1, FN = 9; DeepSeek-R1: TP = 5, FP = 3, FN = 8; Grok-4: TP = 4, FP = 6, FN = 9. Notably, in Group A, Grok-4's Kappa value dropped to −0.54, indicating a negative correlation between its outputs and actual decisions, representing the poorest decision consistency (see Tables 3, 4, and Figure 2a).

**Table 3:** True/false positive classification and consistency tests for each model.

Model group	TP	FP	FN	TN	Kappa Value	* P*
LLM1-A	5	4	8	9	0.08	0.68
LLM1-B	11	1	2	12	0.77	<0.01
LLM2-A	4	6	9	7	−0.15	0.42
LLM2-B	4	1	9	12	0.23	0.14
LLM3-A	6	8	7	5	−0.15	0.43
LLM3-B	5	3	8	10	0.15	0.4
LLM4-A	3	10	10	3	−0.54	0.01
LLM4-B	4	6	9	7	−0.15	0.42

**Table 4:** Spearman correlation between decision confidence and accuracy.

Model Group	*t*	*Sig*
LLM1-A	0.54	0.005
LLM1-B	0.55	0.003
LLM2-A	0.2	0.33
LLM2-B	0.49	0.01
LLM3-A	−0.03	0.9
LLM3-B	0.21	0.3
LLM4-A	0.43	0.02
LLM4-B	0.47	0.01

### 3.3 Correlation analysis between decision confidence and decision accuracy

**Figure 3:**
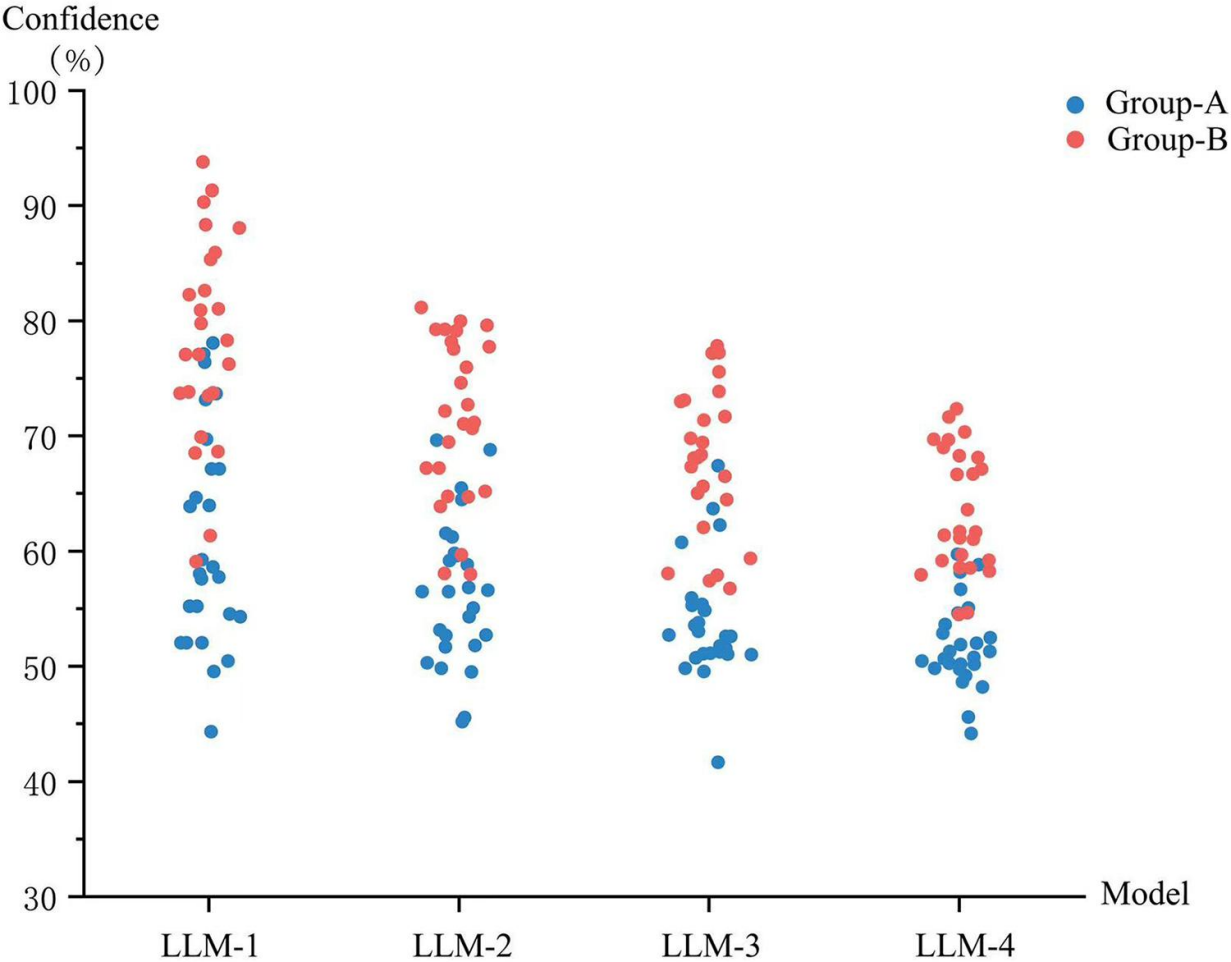
Decision confidence of different LLMs.

See Table 4 and Figure 3.

### 3.4 Performance comparison of different LLMs

After weighting each evaluation metric using the Analytic Hierarchy Process (AHP) and calculating the composite score, GPT-5 achieved the highest score in Group B, at 0.79, significantly outperforming the other models (Gemini 2.5 Pro: 0.52, DeepSeek-R1: 0.46, Grok-4: 0.32). In the information-constrained Group-A, GPT-5 also outperformed other models with a score of 0.42. These multidimensional results further validate GPT-5's relative superiority in FAI treatment decision support tasks (see Table 5 for details).

**Table 5:** Performance comparison of different LLMs.

Model group	Performance weighted score
LLM1-A	0.42
LLM1-B	0.79
LLM2-A	0.32
LLM2-B	0.52
LLM3-A	0.35
LLM3-B	0.46
LLM4-A	0.18
LLM4-B	0.32

## 4 Discussion

The rapid advancement of artificial intelligence technology has driven the widespread adoption of large language models (LLMs), and their applications have progressively expanded into specialized fields such as healthcare, with related research also experiencing rapid growth (23, 24). Among these applications, the potential of LLMs in assisting clinical decision-making has garnered particular attention (25). In clinical practice, healthcare professionals face numerous complex decision-making tasks daily. These decisions are susceptible to influences such as individual experience and cognitive biases, and may lead to misjudgments due to incomplete patient information. Notably, some decisions have a direct impact on patients' long-term prognosis. Consequently, leveraging artificial intelligence to develop personalized, optimized treatment plans based on existing clinical evidence has become a critical research direction for enhancing medical efficiency and safety. However, there is currently no consensus on how to integrate AI tools into clinical practice. Key unresolved research questions include how to provide LLMs with sufficient, structured medical information to maximize their effectiveness and how to systematically evaluate performance differences among various LLMs in real-world clinical settings. Based on this, this study aimed to explore the feasibility and effectiveness of LLMs in supporting treatment decisions for FAI through empirical comparison. While this empirical comparison provides valuable preliminary insights, it is essential to first acknowledge its exploratory nature and inherent limitations, most notably the modest sample size of 26 cases. This limited cohort, although sufficient for hypothesis generation and initial performance comparisons, may affect the stability of the performance metrics and the generalizability of the findings. Therefore, all results and conclusions should be interpreted with caution, serving as a foundation for larger-scale, more definitive studies.

### 4.1 Key findings

This study systematically evaluated and compared the performance of four LLMs (GPT-5, Gemini 2.5 Pro, DeepSeek-R1, and Grok-4) in assisting FAI treatment decision-making. We analyzed LLM outputs across multiple dimensions, including decision rationale (comprehensive analysis of clinical symptoms, disease progression, specialist examination results, imaging findings, occupational/economic factors, and prior treatment history) and decision confidence (expressed as a numerical value accurate to two decimal places). While all four LLMs generated outputs containing rationale, conclusions, and confidence scores, their performance exhibited significant variations. Results indicate that GPT-5 achieved the highest performance across all tasks. Whether relying solely on structured imaging reports (Group-A) or combining structured medical records with structured imaging reports (Group B), GPT-5 demonstrated superior decision accuracy and confidence scores compared to other models, delivering the most outstanding overall performance. The remaining models ranked in descending order of performance as Gemini 2.5 Pro, DeepSeek-R1, and Grok-4. It is noteworthy that this study exclusively employed Chinese instructions for interacting with the LLMs. Even under these conditions, GPT-5 demonstrated a clear advantage, reflecting its robust reasoning capabilities, strong adaptability to the Chinese linguistic context, and superiority in processing medical information.

Specifically, taking GPT-5 as an example, its decision accuracy and confidence in Group B both showed significant improvement compared to Group A. In contrast, Grok-4's outputs for surgical cases in Group A were predominantly “uncertain,” with decision confidence mostly falling within the “low” to “medium” range. In Group B, this model provided more precise decision recommendations (e.g., “recommend surgery” or “do not recommend surgery”), with confidence levels predominantly distributed in the “moderate” range. Overall, all LLMs performed better in Group B than in Group A, indicating that simulated multimodal inputs, which combine structured medical records with radiological text reports, significantly enhance the quality of LLMs' outputs in medical decision-making.

Based on these findings, consulting LLMs solely with chief complaints or symptom descriptions yields limited decision-support effectiveness and fails to provide clinically valuable recommendations. Therefore, when leveraging LLMs to assist in formulating surgical or treatment plans, accurate, detailed, and structured case information should be provided to effectively stimulate their reasoning capabilities and fully realize their potential in clinical decision support. This finding also suggests that the establishing standardized, multimodal clinical data input processes holds significant importance for future clinical practice.

### 4.2 Potential mechanisms behind LLM performance variability

FAI treatment decision-making is inherently complex and challenging, often requiring the balancing of potential risks against anticipated benefits to determine surgical necessity. This decision depends not only on the severity of bone and soft tissue injuries but also on individual patient factors such as age, functional status, expectations, and treatment goals. Consequently, making scientifically accurate decisions in clinical research and practice remains difficult.

Based on the findings of this study, GPT-5 demonstrated the best overall performance in assisting with FAI treatment decision-making tasks, particularly excelling in accuracy and decision confidence, outperforming the other LLMs. The performance ranking was GPT-5, followed by Gemini 2.5 Pro, DeepSeek-R1, and Grok-4. This gradient phenomenon in performance differences likely stems from underlying mechanisms closely related to model architecture design, the quality and breadth of training data, and their adaptability to specific clinical tasks. These factors provide important directions for further optimizing and developing medical applications of LLMs.

#### 4.2.1 Differences in clinical context understanding and multimodal information fusion capabilities

GPT-5's outstanding performance may be attributed to its pre-training and fine-tuning on large-scale, high-quality, and meticulously annotated medical corpora (26). As a generative pre-trained Transformer model (27), GPT-5 leverages the Transformer architecture to build robust semantic understanding and generation capabilities through continuous learning. This enables it to demonstrate deeper semantic parsing when processing complex, unstructured Chinese medical texts (e.g., chief complaints, treatment feedback, and specialty examination records). Crucially, under the Group-B configuration, GPT-5 demonstrates exceptional simulated multimodal information fusion capabilities. It effectively correlates and synthesizes radiological findings with individualized clinical contexts (e.g., age, occupation, insurance type), thereby simulating decision-making processes more closely aligned with those of clinical experts.

In contrast, other LLMs exhibit certain limitations in this regard. Although Gemini 2.5 Pro possesses foundational multimodal processing capabilities, its information fusion strategy tends to be conservative when handling purely text-based structured medical records. It primarily relies on objective MRI findings while underutilizing “soft” yet critical clinical information within the records (e.g., patient feedback on conservative treatment, occupational factors). This results in reduced clinical applicability for decision-making in borderline cases, manifesting as high precision but insufficient sensitivity. DeepSeek-R1, a model emphasizing reasoning (28), demonstrated chain-of-thought reasoning capabilities in this study. However, the stability of its reasoning process may be constrained by the coverage and quality of Chinese medical corpora, which can lead to reasoning biases when encountering specialized terminology. Grok-4's relatively weaker performance may relate to the proportion and quality of medical content in its training data. It demonstrated significant difficulties in understanding specialized imaging terms, such as “labral morphology and signal,” and in associating social determinants, like “insurance type,” with occupation, resulting in lower decision accuracy and specificity. Notably, a study in the field of esophageal cancer also suggests that Grok-4 may have weaker cross-scenario adaptability (29), which consistent with the observations of this study in FAI.

#### 4.2.2 Differences in reasoning depth and decision confidence calibration

GPT-5's reinforcement learning and chain-of-thought reasoning techniques enable it to execute more complex clinical reasoning chains. This structured reasoning process directly enhances the logical consistency of its decisions. Concurrently, our correlation analysis reveals a strong positive relationship between GPT-5's “decision confidence” and its decision accuracy, indicating superior calibration capabilities in decision confidence.

Other LLMs exhibit distinct characteristics in decision confidence vs. reasoning: Gemini 2.5 Pro appears to display “overcautiousness,” with decision confidence consistently falling within the medium range. Even when providing correct answers, it struggles to assign matching high confidence levels, diminishing the reliability of its confidence scores as a clinical reference. DeepSeek-R1 exhibits a “mismatch between confidence and capability.” In specific scenarios, it arrives at erroneous conclusions based on flawed reasoning chains while assigning them high confidence levels. This miscalibration poses a significant risk that requiring heightened vigilance in clinical applications. Grok-4's decision confidence distribution is more dispersed and weakly correlated with accuracy, reflecting instability in its internal decision-making mechanisms.

In summary, success in high-barrier clinical decision support, such as in FAI, relies on high-quality medical data foundations, robust multimodal information fusion capabilities, stable and calibratable reasoning mechanisms, and deep alignment with professional contexts. GPT-5 demonstrates relatively comprehensive advantages across these dimensions, while other LLMs exhibit shortcomings in one or more areas, leading to overall performance disparities.

### 4.3 Analysis of hallucination cases

In this study, we observed that some LLMs generated recommendations inconsistent with final clinical decisions in specific cases, potentially exhibiting the so-called “hallucination phenomenon” (30). Notably, when processing the same medical record with three systems (Gemini 2.5 Pro, DeepSeek-R1, and Grok-4), all recommended surgical treatment in Group A (containing only imaging reports). However, when presented with Group B (which included structured medical record information), they consistently shifted to recommending conservative treatment, with decision confidence levels remaining in the “medium” range. This reversal suggests that LLMs may activate different internal reasoning pathways when exposed to additional information, though it may also amplify inconsistencies in their judgments. This phenomenon was exemplified by case S3, the third patient in the surgical group, as shown in Figure 2a.

Using the S3 case as an example again, this patient ultimately underwent surgery, and an independent third-party assessment confirmed the necessity of the procedure. The imaging report indicated “joint space narrowing,” and the patient was 58 years old. Models like DeepSeek-R1 recommended conservative treatment based on the widely accepted clinical knowledge that “joint space narrowing is a relative contraindication for hip arthroscopy.” This demonstrates that LLMs can identify and apply authoritative medical consensus, with reasoning grounded in reasonable medical rationale to some extent. However, real-world clinical decisions often integrate nuanced considerations across multiple dimensions. In this case, the patient actually had only mild joint space narrowing. Factors such as functional requirements, pain severity, response to conservative treatment, and shared decision-making between physician and patient likely contributed to the clinical justification for proceeding with surgery. Such nuanced information—often existing in unstructured forms or embedded within doctor-patient communication—remains difficult for current text-based, structured inputs to capture fully. Therefore, this case should be viewed as a representative example highlighting the gap between LLM decision logic and the complexity of real clinical decision-making, rather than merely a “hallucination.” It highlights that even when LLMs reason based on sound medical knowledge, their outputs may diverge from reality if they cannot access, quantify, or integrate the nuanced contextual information influencing clinical decisions. This suggests future research should focus on developing more refined, clinically relevant multimodal input frameworks and advancing LLMs' contextual weighing and comprehensive judgment capabilities. Although this inconsistency occurred in only one case within the study sample, indicating that current LLMs remain generally reliable, the limitations revealed warrant vigilance in clinical support applications. Physicians and patients using such tools should understand that their recommendations stem from limited structured information, and that final treatment plans must incorporate comprehensive clinical assessment and professional judgment, in the real world.

### 4.4 Clinical applicability and potential risks

Based on these findings, we conclude that LLMs demonstrate high feasibility and significant potential in assisting treatment decisions for FAI. Current treatment decisions for this condition are challenging due to its complex and varied symptoms, coupled with uncertain surgical outcomes for some patients. Compared to surgical decisions for common hip conditions, such as hip fractures or avascular necrosis of the femoral head, FAI treatment decisions present greater complexity. LLMs can comprehensively analyze patient medical histories and imaging reports, reducing the likelihood of clinical information omission. By conducting holistic assessments of patient conditions, healthcare providers can make more comprehensive and evidence-based decisions.

However, it is crucial to note that LLM performance is highly dependent on the completeness of input data. Insufficient or biased input information may lead to erroneous decisions. For instance, patient age and the specific assessment of joint space narrowing and labral tear severity in imaging reports directly impact the accuracy of decisions. In this study, limitations in input data specifically triggered the “hallucination” phenomenon, introducing bias in the decision between “conservative treatment” and “surgical intervention.” Therefore, to maximize the utility of LLMs, comprehensive and detailed patient information must be provided to ensure accurate interpretation of clinical evidence. Additionally, attention must be paid to the potential for LLMs to exhibit an over-tendency toward surgical recommendations based on characteristics of specific patient groups (e.g., younger individuals or those with better economic conditions). Consequently, in practical applications, medical professionals should remain vigilant regarding LLM outputs, combining them with extensive clinical experience to inform final decision-making and avoid risks associated with sole reliance on LLMs.

### 4.5 Comparison with previous studies

LLMs have been progressively applied in orthopedics, but we observe relatively few studies focusing on their use for FAI. Adelstein et al. (31) focused on the responses of LLMs to common patient questions about FAI. Slawaska-Eng et al. (32) demonstrated that two versions of ChatGPT provided relatively accurate responses to common questions related to FAI and arthroscopic surgery, with no significant differences between the versions. They noted its potential value in patient education but did not address its actual clinical application in decision-making. Chen et al. (33) summarized LLM responses to FAI-related questions but did not conduct comparative analyses between LLMs. This study represents a significant innovation in the specialized field of FAI. It is the first to utilize a Chinese-language LLM for diagnostic and treatment decisions in FAI as an experimental scenario, establishing a comprehensive evaluation chain that encompasses structured case data and MRI image descriptions. This work fills a gap in LLM decision support research within the specialized context of FAI and the Chinese language.

### 4.6 Methodological reflections and limitations

Although this study pioneered a comparative analysis of LLM performance in FAI diagnosis and treatment decision-making through standardized clinical records and a systematic evaluation protocol, several limitations warrant reflection and point to future research directions.

First, the data originated from a single leading orthopedic specialty hospital in China. While this ensured diagnostic accuracy and treatment standardization, it also introduces inherent limitations. The single-center design means that patient demographics, clinical workflows, and physician decision-making preferences may reflect institution-specific characteristics, thereby limiting the generalizability of findings to broader populations, healthcare facilities of varying levels, or different healthcare systems. Moreover, the reference standard used in this study—the final treatment decision made at our institution—does not represent an absolute clinical truth, as treatment choices for FAI can be influenced by institution-specific factors such as surgical preferences, rehabilitation resources, and shared decision-making with patients. Consequently, the performance metrics reported primarily reflect alignment with the decision-making patterns of our center rather than an objective measure of clinical appropriateness. Future studies should validate the robustness of LLM-assisted decision support across multiple centers and geographic regions, ideally using externally validated expert consensus as a reference standard.

Second, to control variables, we rigorously structured and standardized medical records and MRI reports. While this was a necessary methodological choice, it inevitably resulted in the loss of some information from the original clinical data. Orthopedics is a visually intensive specialty that relies heavily on the intuitive interpretation of imaging studies. In real-world practice, physician decision-making often relies on subtle linguistic cues within free-text descriptions, intuitive features inherent to the images themselves, and information gleaned from physician-patient communication—elements that are difficult to structure and analyze thoroughly. Therefore, this study primarily evaluates the processing capabilities of LLMs for “standardized clinical information,” which may overestimate or underestimate their performance in fully authentic, real-world clinical settings. Future research should explore the capacity of LLMs to integrate multimodal information streams that more closely to resemble real-world scenarios, including the images themselves.

Third, this study employed a fixed, optimized prompt framework to ensure consistency in evaluation. However, such static prompts may not fully unlock the potential of all LLMs. Different LLMs may exhibit varying sensitivities to subtle variations in prompts, such as role-setting or task-decomposition instructions. Despite multiple rounds of optimization, the current framework may be better suited to the response patterns of certain LLMs. Future research could explore dynamic or interactive prompting strategies to uncover the optimal performance of various LLMs more equitably.

Fourth, the field of artificial intelligence, particularly LLMs, evolves at a rapid pace. The LLM versions evaluated in this study are time-specific. Continuous updates and iterations by vendors may rapidly alter model performance, meaning our conclusions represent a “snapshot” of capabilities at a specific point in time, requiring follow-up studies for tracking and validation. Furthermore, this research focuses on “black-box” evaluation of decision outputs without delving into the underlying logic or interpretability of LLM decision-making. This represents an important future research direction integrating explainable AI technologies.

### 4.7 Clinical and research implications

The findings of this study transcend mere performance rankings of LLMs, offering substantial implications for future orthopedic clinical practice and interdisciplinary research between medicine and engineering.

#### 4.7.1 Implications for clinical practice

##### 4.7.1.1 As efficient clinical decision support tools

Research indicates that high-performing LLMs (e.g., GPT-5) have the potential to serve as efficient auxiliary tools for residents or primary care physicians. They can rapidly synthesize patient information and provide evidence-based decision references, helping reduce decision variability caused by inexperience or incomplete information.

##### 4.7.1.2 Redefining patient education and consultation models

This study lays the groundwork for developing specialized FAI patient education and Q&A templates. These templates guide patients to use optimized prompts when consulting reliable LLMs, enabling them to access accurate, systematic disease and treatment knowledge. This approach alleviates pre-consultation anxiety and enhances the efficiency of doctor-patient communication.

##### 4.7.1.3 Defining current application boundaries

The study also clearly indicates that even the best LLMs are not infallible in their decisions, and confidence calibration requires further refinement. Therefore, at this stage, LLMs must be strictly positioned as “aids” rather than “replacements.” Their outputs must undergo final review and decision-making by the attending physician, taking into account clinical examinations, imaging interpretations, and patient preferences.

#### 4.7.2 Recommendations for future research

To advance real-world prospective validation, our research team plans to move beyond retrospective case repositories by conducting prospective randomized controlled trials that integrate LLM decision-making into actual clinical workflows. This will evaluate their long-term impact on physician decision-making efficiency and patient outcomes.

Deepening multimodal fusion and interpretability research: This study confirms the value of LLMs in simulated multimodal scenarios, utilizing radiological text reports and textual medical records. Future work on decision support should explore the development of accurate multimodal models that directly process DICOM-formatted MRI images while integrating natural language descriptions. Concurrently, interpretable AI technologies must be introduced to reveal the key features underpinning LLM decisions (e.g., demographics such as whether based on imaging morphology or patient age and occupation) to enhance clinician trust.

AS outlined above, this study marks a significant step toward systematically applying LLMs to the complex decision-making involved in treating FAI. It not only provides evidence for clinicians to identify more reliable LLM tools, but, more importantly, paves the way for the future development of human-machine collaborative, efficient, and precise orthopedic intelligent diagnosis and treatment.

## 5 Conclusions

This study systematically compared and analyzed the performance of four mainstream LLMs in the task of assisting FAI treatment decision-making. The core conclusion indicates that at the current stage of technological development, LLMs have demonstrated significant potential to become powerful auxiliary tools for orthopedic surgeons. However, their performance varies considerably, and they have not yet reached the level of maturity required to replace human professional judgment fully.

Specifically, GPT-5 demonstrated robust, comprehensive decision-making capabilities within the Chinese clinical context defined in this study. Its advantages lie in the deep integration of multimodal clinical information, stable chain-of-thought reasoning, and superior decision confidence calibration, making it currently the closest to meeting clinical assistance requirements—though it remains positioned solely at the auxiliary decision-making level. Gemini 2.5 Pro and DeepSeek-R1 followed closely, each demonstrating distinct strengths in handling structured data rigor and complex reasoning potential, respectively. Both exhibited identifiable shortcomings in specific areas such as contextual understanding or confidence calibration. Grok-4's performance highlighted the challenges faced by general-purpose LLMs when tackling specialized tasks without sufficient domain-specific knowledge alignment. Given the study's preliminary nature, characterized by a small, single-center, retrospective design, the results should be interpreted with caution. Future large-scale, prospective, multi-center studies are essential to validate these findings and assess the clinical safety and generalizability of LLM-assisted decision support.

## Data Availability

The raw data supporting the conclusions of this article will be made available by the authors, without undue reservation.
